# Obesity – an inflammatory state

**DOI:** 10.1186/1751-0147-57-S1-K5

**Published:** 2015-09-25

**Authors:** Isabelle Wolowczuk

**Affiliations:** 1 Centre National de la Recherche Scientifique (CNRS), Pasteur Institute of Lille, Lille, France

## 

Obesity is increasing at an alarming rate, with significant negative health, social, and economic consequences. While its impact on metabolism is manifest in the development of insulin resistance and type 2 diabetes, there are also connections between obesity and several chronic inflammatory diseases. Indeed, obese individuals are at higher risk of cancer, asthma, as well as intestinal and pulmonary diseases and their overall mortality is higher. Chronic low-grade inflammation in the expanding white adipose tissue (WAT) may mediate the association between excessive body fat accumulation and inflammatory diseases. The exact trigger of WAT inflammation is unknown, however, several mechanisms have been proposed such as e.g. hypoxia, endoplasmic reticular stress, and excess saturated fatty acids. Furthermore, WAT macrophages and T lymphocytes have also important roles in the inflammatory process: proinflammatory macrophages (M1 macrophages) accumulated in the obese WAT while in contrast the numbers and frequencies of anti-inflammatory regulatory T cells (Tregs) and group 2 innate lymphoid cells (ILC2) are reduced. Finally, alterations in gut microbiota composition and diversity were recently suggested to play a key role in the development of obesity-related inflammatory diseases. Our talk will provide an update on the mechanistic aspects of the pathogenesis of adipose tissue inflammation, insulin resistance and chronic inflammatory diseases such as those resulting from pulmonary infections. We will also suggest some new therapeutic strategies that could be derived in order to prevent and/or reverse these processes; such as the development of immunological or dietary interventions.

